# Usability, acceptability, and feasibility of the World Health Organization Labour Care Guide: A mixed‐methods, multicountry evaluation

**DOI:** 10.1111/birt.12511

**Published:** 2020-11-22

**Authors:** Joshua P. Vogel, Liz Comrie‐Thomson, Veronica Pingray, Luis Gadama, Hadiza Galadanci, Shivaprasad Goudar, Rose Laisser, Tina Lavender, David Lissauer, Sujata Misra, Yeshita Pujar, Zahida P. Qureshi, Taiwo Amole, Mabel Berrueta, Faisal Dankishiya, George Gwako, Caroline S. E. Homer, Jonathan Jobanputra, Sam Meja, Carolina Nigri, Vandana Mohaptra, Alfred Osoti, Javier Roberti, Dennis Solomon, Maryam Suleiman, Gianna Robbers, Shireen Sutherland, Sunil Vernekar, Fernando Althabe, Mercedes Bonet, Olufemi T. Oladapo

**Affiliations:** ^1^ Maternal, Child and Adolescent Health Program Burnet Institute Melbourne Victoria Australia; ^2^ Department of Epidemiology and Preventive Medicine Monash University Melbourne Victoria Australia; ^3^ Faculty of Medicine and Health Sciences Ghent University Ghent Belgium; ^4^ Department of Mother and Child Health Research Institute for Clinical Effectiveness and Health Policy (IECS‐CONICET) Buenos Aires Argentina; ^5^ College of Medicine Department of Obstetrics and Gynaecology University of Malawi Zomba Malawi; ^6^ Africa Center of Excellence for Population Health and Policy Bayero University Kano State Nigeria; ^7^ KLE Academy of Higher Education and Research J N Medical College Belagavi Karnataka India; ^8^ Archbishop Antony Mayala School of Nursing Catholic University of Health and Allied Health Sciences Mwanza Tanzania; ^9^ Division of Nursing, Midwifery and Social Work School of Health Sciences Faculty of Biology, Medicine and Health The University of Manchester Manchester UK; ^10^ Malawi‐Liverpool‐Wellcome Trust Research Institute Queen Elizabeth Central Hospital College of Medicine Blantyre Malawi; ^11^ Institute of Lifecourse and Medical Sciences University of Liverpool Liverpool UK; ^12^ Fakir Mohan Medical College & Hospital Balasore Odisha India; ^13^ Department of Obstetrics and Gynaecology School of Medicine University of Nairobi Nairobi Kenya; ^14^ Queen Elizabeth Central Hospital Blantyre Malawi; ^15^ Thyolo District Hospital Thyolo Malawi; ^16^ Muhammad Abdullahi Wase Teaching Hospital Kano Nigeria; ^17^ Development and Research Training in Human Reproduction (HRP) Department of Sexual and Reproductive Health and Research UNDP/UNFPA UNICEF/WHO/World Bank Special Programme of Research World Health Organization Geneva Switzerland

**Keywords:** childbirth, intrapartum care, labor, partograph, WHO Labour Care Guide

## Abstract

**Introduction:**

The World Health Organization’s (WHO) Labour Care Guide (LCG) is a “next‐generation” partograph based on WHO’s latest intrapartum care recommendations. It aims to optimize clinical care provided to women and their experience of care. We evaluated the LCG’s usability, feasibility, and acceptability among maternity care practitioners in clinical settings.

**Methods:**

Mixed‐methods evaluation with doctors, midwives, and nurses in 12 health facilities across Argentina, India, Kenya, Malawi, Nigeria, and Tanzania. Purposively sampled and trained practitioners applied the LCG in low‐risk women during labor and rated experiences, satisfaction, and usability. Practitioners were invited to focus group discussions (FGDs) to share experiences and perceptions of the LCG, which were subjected to framework analysis.

**Results:**

One hundred and thirty‐six practitioners applied the LCG in managing labor and birth of 1,226 low‐risk women. The majority of women had a spontaneous vaginal birth (91.6%); two cases of intrapartum stillbirths (1.63 per 1000 births) occurred. Practitioner satisfaction with the LCG was high, and median usability score was 67.5%. Practitioners described the LCG as supporting precise and meticulous monitoring during labor, encouraging critical thinking in labor management, and improving the provision of woman‐centered care.

**Conclusions:**

The LCG is feasible and acceptable to use across different clinical settings and can promote woman‐centered care, though some design improvements would benefit usability. Implementing the LCG needs to be accompanied by training and supportive supervision, and strategies to promote an enabling environment (including updated policies on supportive care interventions, and ensuring essential equipment is available).

## 1 INTRODUCTION

In the past two decades, considerable efforts have been made to encourage and support pregnant women to give birth in health facilities, where they ideally receive good‐quality intrapartum care from skilled health personnel. An essential component of this care is ensuring that women are adequately monitored during labor, typically by prospectively completing a partograph based on regular clinical assessments during labor.^1^ Although partograph designs vary, they typically track a woman’s vital signs, cervical dilatation, fetal descent, uterine contractions, the use of medications in labor, and fetal well‐being. The WHO‐modified partograph also has alert and action lines, which were meant to trigger interventions during labor if progress is slower than 1 cm per hour.^1^ In February 2018, WHO published new recommendations on intrapartum care for a positive childbirth experience, which included updated definitions and durations for first and second stage of labor, based on evidence from systematic reviews.^2^, ^3^, ^4^ The WHO guideline panel concluded that the duration of the first and second stage of labor is highly variable, and a cervical dilation rate of 1 cm per hour in the first stage is unrealistically fast for some women. They also concluded that a cervical dilatation rate slower than 1 cm per hour was by itself a poor predictor of adverse birth outcomes, and should not be the sole indication for obstetric intervention.^2^

To help practitioners implement these recommendations, WHO subsequently initiated development of a “next‐generation” partograph known as the WHO Labour Care Guide (LCG) (Box 1, File S1). The new design was intended to promote woman‐centered care, stimulate practitioners to think critically around labor decision‐making, and (to the extent possible) individualize labor monitoring. Development included expert consultations, iterative prototype development and testing, and international survey of maternity care practitioners and qualitative research with midwives from 6 African countries. New features included the following: monitoring the use of supportive care interventions (such as companionship and pain relief); removing the 1 cm per hour threshold relating to the alert and action lines; documenting numerical values for monitored parameters; and explicit threshold values to trigger action following all maternal and fetal assessments.

**Aims of the WHO Labour Care Guide**

The Labour Care Guide aims to support health care practitioners:

- Monitor maternal and fetal status and progress in labor;
- Continuously remind practitioners to offer supportive care throughout labor;
- Guide practitioners on what observations should be regularly made and recorded during labor, to identify any emerging complication in mother and/or baby;
- Provide reference thresholds for abnormal labor observations that should trigger specific action(s);
- Minimize overdiagnosis and underdiagnosis of abnormal labor events and unnecessary use of interventions; and
- Support audit and quality of care improvement.

It is critical to ensure that the LCG performs as intended and can meet the needs of maternity care practitioners. The aim of this project was to evaluate the usability, feasibility, and acceptability of the LCG, and barriers and facilitators to its use, in routine clinical settings. It also aimed to identify what improvements could be made to the LCG to enhance usability.

## 2 METHODS

This was a three‐phased, mixed‐methods project where skilled health personnel (ie, doctors, midwives, or nurses) were trained in how to use the LCG (Phase 1), applied the LCG in managing the labor of low‐risk women (Phase 2), and participated in focus group discussions on their experiences (Phase 3). We adopted the International Organization for Standardization definition of usability as “the extent to which a product can be used by specified users to achieve specified goals with effectiveness, efficiency and satisfaction in a specified context of use.”^5^ The LCG design used in this evaluation is provided (File S1). The project was conducted in accordance with Good Clinical Practice (GCP) standards, and results reported were in line with STROBE and COREQ guidance.^6^, ^7^ All participating practitioners and women signed an informed consent form before participation.

### 2.1 Setting

A convenience sample of 12 hospitals across six countries (Argentina, India, Kenya, Malawi, Nigeria, and Tanzania) was identified. Participating hospitals were those with more than 1000 births per year, with a minimum of 25 skilled health personnel working in labor ward, and who were able to provide at least basic emergency obstetric and neonatal care (Table S1). Apart from the LCG, clinical care provided to women was according to the existing practices in these hospitals.

### 2.2 Design and participants

#### 2.2.1 Phase 1—health care practitioner sampling and training

At each hospital, at least 10 skilled health personnel who were employed on labor ward and experienced in use of a partograph were approached to participate. Practitioners were purposively sampled to ensure participation of relevant cadres providing labor care in the hospital, and representation from senior and junior staff and from different shifts. Consenting practitioners attended an in‐person workshop where they received practical training on how to use the LCG correctly, by means of a standardized implementation manual and training package in English (five countries) and Spanish (Argentina only).

#### 2.2.2 Phase 2—recruitment of women and application of LCG

Women attending participating hospitals for childbirth were prescreened in order of arrival to identify women being admitted for childbirth with term, singleton pregnancies with a live fetus and normal vital signs. These women were formally screened using a standardized form. If eligible, they were invited to undergo an informed consent process by means of a private interview in a language of the woman’s choice. Eligible women were aged 18‐34 years; at ≤5 cm cervical dilatation with a cephalic presentation and a vaginal birth was anticipated; had no uterine scar; and were otherwise considered as low‐risk according to the local guidelines. Consenting women were enrolled and their data collected until 1 hour after complete expulsion of the placenta. Practitioners continued to enroll women until the per‐country sample size of 200 women was reached.

#### 2.2.3 Phase 3—focus group discussions with practitioners

In Phase 3, all participating practitioners were invited to attend a focus group discussion (FGD) and completed a questionnaire on their experiences with the LCG. One FGD per hospital was conducted using a structured discussion guide (File S2) and led by a local facilitator experienced in qualitative research methods. The FGDs explored health care practitioners’ views on usability, feasibility, and acceptability, and barriers and facilitators to using LCG in their setting. Participants were also invited to suggest improvements to the LCG design and training materials. In five of the six countries (India, Kenya, Malawi, Nigeria, and Tanzania), FGDs were conducted in English or a mix of English and local language. In Argentina, FGDs were conducted in Spanish.

### 2.3 Data collection and management

Participating practitioners and enrolled women were assigned unique numbers, and only deidentified data were collected. Each practitioner completed a questionnaire after the initial training workshop. Practitioner’s experiences with LCG were collected by means of a postpartum questionnaire after each birth using a 5‐point Likert scale. After enrollment had concluded, each practitioner completed a satisfaction and usability questionnaire. Satisfaction was measured for each section of the LCG and overall using a 5‐point Likert scale. Usability was assessed using an adaptation of the System Usability Scale (SUS), a valid, reliable, and widely used 10‐item instrument for assessing usability.^8^ Project staff also extracted data from medical records on enrolled women’s characteristics, the use of obstetric interventions during labor and childbirth, and birth outcomes. All data were collected using paper forms and entered into REDCap,^9^ a GCP‐compliant, password‐protected online data management system with validation checks. Data queries and discrepancies were resolved at site level. All project‐related information was stored securely at project sites, with participant information in locked file cabinets in areas with limited access.

### 2.4 Analysis

#### 2.4.1 Quantitative analysis

All data were generated from the online data management system, with the analysis based on the multicountry database. The analysis was descriptive (without statistical inference testing), with outcomes reported by site and overall. Categorical data were reported using proportions and percentages, and continuous variables, using median and interquartile range (IQR). Data on practitioner’s perspectives were assessed overall and by site, cadre (doctor, midwife, nurse), maternal parity (nulliparous or multiparous), and number of births attended using LCG (<5 or ≥5 births). SUS scores were converted to a score out of 100, with >70 considered “good” (File S3).^10^ Analyses were performed using SPSS 26 (IBM Corp).

#### 2.4.2 Qualitative analysis

All FGD transcripts, field notes, and participant‐generated data were imported into NVivo 12 (QSR International). Transcripts in Spanish were translated into English, with coding of all transcripts conducted centrally. Qualitative data were analyzed using framework analysis.^11^ An *a priori* coding framework was developed based on two existing evidence syntheses exploring barriers and enablers for partograph use.^12^, ^13^ Two researchers applied these codes in duplicate to two transcripts from one country (Tanzania), and compared data under each code to refine the coding framework and code definitions. The final coding framework was applied independently by the researchers to the remaining 10 transcripts. Researchers met frequently during coding to share reflections on the data and discuss application of the coding framework. Once coding was complete, data under each code were reviewed, and axial coding was applied to develop subcodes and analytical themes. Qualitative research leads from each site verified country‐specific data contributing to each finding and contributed to overall interpretation.

## 3 RESULTS

### 3.1 Quantitative findings

A total of 136 practitioners across 12 hospitals participated. In total, 64.7% were midwives, 33.1% were doctors, and 2.2% were nurses (Table 1). After completing the practical training, most practitioners agreed or strongly agreed that the workshop and the training manual were helpful (95.1%) and that they felt ready to use the LCG (82.4%) (Table S2). Participating practitioners identified 1268 eligible women, of whom 1,226 consented and were enrolled. Most women were aged between 18 and 30 years (85.1%), married or cohabitating (95.4%), had 10 or more years of formal education (61.6%), and had no previous births (43.4%) or 1‐2 previous births (41.8%) (Table 2). In total, 91.6% had a spontaneous vaginal birth, 1.3% had an instrumental vaginal birth, and 7.1% had a caesarean section. In total, 4.4% of babies were <2500 g, 3.3% were more than 4000 g, and there were two stillbirths (equating to an intrapartum stillbirth rate 1.63 per 1000 births). No maternal deaths occurred.

**Table 1:** Characteristics of participating practitioners

	N	%
Total	136	100.0
Professional role		
Doctor	45	33.1
Midwife	88	64.7
Nurse	3	2.2
Number of years of experience providing clinical care in labor ward		
≤1 yr	26	19.1
<1‐5 yrs	60	44.1
>5 yrs	50	36.8
Gender		
Female	99	72.8
Male	37	27.2
Births attended in the past 4 wks^a^		
Less than 30 births	84	61.8
30 to < 50 births	18	13.2
50 or more births	34	25.0

^a^ Prior to commencement of project.

**Table 2:** Characteristics of participating women

	N	%
Total	1226	100.0
Maternal age (y)		
18‐24	616	50.2
25‐30	428	34.9
31‐34	182	14.8
Maternal education (y)		
0‐6	177	14.4
7‐9	294	24.0
10‐12	500	40.8
13 or more	255	20.8
Marital status		
Married/cohabitating	1170	95.4
Single/separated/widowed/divorced	55	4.6
Missing	1	0.1
Parity		
0	532	43.4
1 or 2	513	41.8
3 or more	181	14.8
Best obstetric estimate of gestational age (completed weeks) at time of birth (median, IQR)	39	2
Final mode of birth		
Cephalic vaginal birth	1113	90.8
Breech vaginal birth	10	0.8
Vacuum or forceps vaginal birth	16	1.3
Cesarean section	87	7.1
Pain relief^a^		
Nonpharmacological pain relief	544	44.4
Oral pain relief medication	47	3.8
Epidural and/or spinal	63	5.1
Injectable analgesic (IM or IV)	187	15.3
No pain relief used	446	36.4
Sex of baby		
Female	626	51.1
Male	600	48.9
Vital status at birth		
Live birth	1224	99.8
Stillbirth	2	0.2
Apgar score at 5 min (1224 liveborns only)		
<7	12	1.0
≥7	1212	99.0
Birthweight		
<2500 g	54	4.4
2500‐4000 g	1132	92.3
>4000 g	40	3.3
Major malformation identified at birth		
No	1224	99.8
Yes	1	0.1
Unknown	1	0.1

^a^ Does not total to 100% as women may have >1 pain management option

When completing questionnaires about their experiences with the LCG after each birth (n = 1226), practitioners agreed or strongly agreed that they were able to use the LCG (96.0%) and complete it correctly (94.2%), that they were satisfied using the LCG (91.0%), and that the LCG was helpful in managing the woman’s labor and childbirth (90.2%) (Table 3). At 79.5% of births, practitioners agreed or strongly agreed that they were satisfied with the LCG design (Table 3). In general, Argentinean practitioners had lower experience rankings than other countries (Table S3). Practitioner experiences were largely similar when stratified by cadre (doctors, midwives, nurses) or woman’s parity; however, there was a trend toward better practitioner experiences when they had attended more than five births using LCG (Table S3).

**Table 3:** Practitioner’s perspectives on using the LCG (after each birth)

	N	%
Total	1226	100
I was able to use the LCG in managing this labour and childbirth		
Strongly agree	580	47.3
Agree	597	48.7
Neither agree nor disagree	46	3.8
Disagree	2	0.2
Strongly disagree	0	0.0
Missing	1	0.1
I was able to complete the LCG correctly		
Strongly agree	495	40.4
Agree	659	53.8
Neither agree nor disagree	57	4.6
Disagree	14	1.1
Strongly disagree	0	0.0
Missing	1	0.1
I was satisfied using the LCG in managing this woman’s labour and childbirth		
Strongly agree	479	39.1
Agree	627	51.1
Neither agree nor disagree	108	8.8
Disagree	11	0.9
Strongly disagree	0	0.0
Missing	1	0.1
The LCG was helpful in managing this woman’s labour and childbirth		
Strongly agree	516	42.1
Agree	600	48.9
Neither agree nor disagree	95	7.7
Disagree	14	1.1
Strongly disagree	0	0.0
Missing	1	0.1
Overall, I am satisfied with the current design of the Labour Care Guide		
Strongly agree	410	33.4
Agree	565	46.1
Neither agree nor disagree	199	16.2
Disagree	47	3.8
Strongly disagree	4	0.3
Missing	1	0.1

After enrollment was completed, each practitioner rated their satisfaction with different sections of the LCG (Table S4). The highest practitioner satisfaction score was reported for the supportive care monitoring section (87.5%), and the lowest score was reported for the birth outcomes section (66.2%). In their final assessment, 75.7% of practitioners agreed they were satisfied with the LCG design overall. Usability scores ranged from 22.5 to 95, with a median score of 67.5 (interquartile range: 57.5 to 75.0). In total, 58 practitioners (42.6%) scored the LCG as 70 or greater (ie, good usability). Within the component questions of the SUS score (File S3), an outlier was 45.5% of practitioners agreeing that “I needed to learn a lot of things before I could get going with the Labour Care Guide.”

### 3.2 Qualitative findings

Twelve FGDs were conducted with 105 staff (35 doctors, 69 midwives, and one nurse). A summary of findings is presented below.

#### 3.2.1 Acceptability

Although many practitioners across all sites found adopting a new tool challenging, most reported that they became comfortable using the LCG with practice.

After the first day […] things started getting easier, and even enjoyable to use after a week (Midwife, Kenya)

There was a consensus that recording numerical observations within the LCG was more precise, and consequently more accurate, than recording information graphically (such as in alternative partographs). However, some practitioners found this required more attention and could be more time‐consuming.

[E]specially while marking the blood pressure and pulse it is easy to write rather than searching for the number and then putting the dot and then connecting the graph which we usually do […] Contractions and the vital [signs] are easy to write. (Doctor, India)

Practitioners in Argentina and Kenya noted that the LCG presented numerical observations in a visual way (by circling alerts, and through documenting dilatation and descent on a grid) and that this was easy to interpret quickly. Other practitioners (in India, Malawi, Nigeria, and Tanzania) said that the LCG is more difficult to quickly scan and interpret, compared with partographs that use predefined alert and action lines.

[T]he previous one [partograph] would tell if the baby is in distress by just looking, it was not taking us too much energy to think that the baby is in distress. Unlike this one, you have to think about how the fetal rate is as compared to the normal. (Midwife, Malawi)

Practitioners across all sites appreciated the one‐page structure and found its content to be streamlined and coherent, though there was a strong consensus that the layout needed to be larger. They consistently reported that the current layout made it impossible to complete legibly in real time. This affected its usefulness, with practitioners not writing all observations, taking too long to write (at the expense of other tasks), writing on a separate piece of paper to copy over later, or writing illegibly.

[O]ne extremely difficult thing is the space to fill. It's tiny. With my handwriting, when I write the heart rate, it cannot be read directly. (Midwife, Argentina)

Practitioners in multiple sites also reported that the frequency of monitoring was different from their usual practice, although several practitioners also noted that it was no more frequent than existing recommendations. In Argentina, practitioners indicated women were concerned that monitoring was less frequent than they expected, whereas in other sites, the frequency of monitoring led some women and families to believe there was a problem. Several practitioners reported that responding to these concerns added to their workload.

#### 3.2.2 Utility

Practitioners reported that the LCG supported frequent, meticulous, and timely monitoring of woman and baby, and guided objective, data‐driven decision‐making, with positive impacts on the quality of labor care and maternal and newborn health outcomes. In all settings except Argentina, practitioners stated the LCG prompted staff to assess women in labor more frequently, and record more observations, compared with routine practice in their labor wards. Practitioners perceived that this had improved quality of care and health outcomes. Specifically, practitioners across multiple sites perceived that the LCG supported them to be more responsive to women’s unique labors, reducing unnecessary and early interventions.

Using the LCG, we show more care to our patients than before, we provided them what they needed. So we had less complications and less interventions [due to] early detection and decision making. (Midwife, Nigeria)

Practitioners across all sites also emphasized that using the LCG guided them to provide supportive, person‐centered labor care—whether or not they had been familiar with WHO guidance on supportive care—and strengthen the relationships between health practitioners and women.

We all know what the WHO's [supportive care] recommendation is, but many professionals do not offer it because they are used to working fast […] No one wants to fill it [the LCG] up with pure no, no, no, so it forces you somehow to offer it [supportive care] to the patient. (Midwife, Argentina)

Overall, practitioners across all sites considered the LCG an effective source of information that was precise, coherent, and relevant. They also reported there was no duplication of information within the LCG, although in some sites, the LCG captured information that was documented in existing local health records. However, some practitioners across multiple sites were concerned that specific information, which is normally reported in the woman’s medical record but which they consider essential to labor care was missing from the LCG. Examples were maternal HIV status, maternal blood type and rhesus status, and postpartum hemorrhage in the third stage of labor.

As you know, HIV now is a challenge in our country […] But here it’s not clearly indicated that the serostatus of the mother is reactive, or how long she has been on treatment, whilst in previous labour charts we were using it’s there. (Midwife, Malawi)

Many practitioners across multiple sites commented that the LCG supported practitioners’ critical thinking in planning labor care and also facilitated clinical handover. Practitioners speculated that staff at primary care facilities (with less training and support) may not be equipped to lead labor care planning.

### 3.3 Anticipated barriers and facilitators to using LCG

Practitioners were asked to consider what possible barriers and facilitators may affect using LCG (based on their initial experience with the LCG and their routine partograph).

#### 3.3.1 Time and workload

Practitioners across all sites reported that heavy workloads in labor wards make it challenging to consistently take all observations and can contribute to making errors. In all sites except Argentina, practitioners indicated that completing the LCG for some women would mean that other women in labor would not receive the care they needed. Practitioners in several sites described situations where they had not been able to complete the LCG, or not in real time, because another woman required urgent or critical care. However, practitioners acknowledged that heavy workloads and time pressures make it challenging to use any partograph, regardless of design.

It is difficult where one staff member has 10 mothers. It is quite difficult, almost impossible, the monitoring will be not be done well. […] (Midwife, Kenya)

Practitioners also commented that the frequency of observations and the time required to make certain observations for the LCG created an additional time burden, compared with their existing partograph. However, practitioners across several sites commented that because the LCG reduced unnecessary and early interventions through improved monitoring and facilitated more responsive, personalized labor care, it reduced their workload overall.

[W]hen we talk about the time that we need to sit and fill it, then we may feel it added to our workload. But eventually, since at the long run, interventions have been reduced and the patients end up with a normal labour and go home after a few hours after delivery instead of having a [caesarean section], and then also staying for more than 4‐5 days […] I think in the long run, it reduced our workload. (Doctor, Nigeria)

#### 3.3.2 Equipment and supplies

In all sites except Argentina, practitioners reported challenges in accessing basic equipment and supplies, such as thermometers, sphygmomanometers, urinalysis strips, watches, and Doppler fetal monitors.

I got challenge for […] filling this protein plus or albumin. […] The resources are for high‐risk women, not every mother in the labour ward. […] they are there for those with oedema and high blood pressure. (Doctor, Tanzania)

#### 3.3.3 Knowledge and skills

Practitioners in multiple sites reported that not all staff responsible for completing the LCG, particularly at primary facilities and in rural areas, would have the skills required to take certain measurements (such as assessing caput, molding, and fetal position), particularly at primary facilities and in rural areas.

Although some practitioners commented that the alerts effectively triggered action, others felt that the absence of alert and action lines made it more challenging to understand how and when to respond, and that a higher level of knowledge would be required to use the LCG.

[I]n the previous partograph […] by just looking at it we know whether it is crossing the alert line […] we can look at it and say that this patient needs immediate intervention, but now it becomes little bit cumbersome to go through the details (Doctor, India)

Practitioners also commented that there needs to be increased knowledge of current WHO labor care recommendations across a range of stakeholders, including health staff working outside the labor ward, health policymakers, and women’s families and communities, in order to support effective use of the LCG.

#### 3.3.4 Policy and procedures

In all countries except Argentina, practitioners reported that national or hospital policies relating to the physical layout of labor wards, equipment and medical supplies, and standard protocols for labor care would need to be updated to provide an enabling environment. This was particularly the case for supportive care interventions requiring pain management protocols, storage of analgesics, and physical arrangements to facilitate birth companions.

[O]ur labour [ward] cannot accommodate and will never accommodate a companion (Midwife, Malawi)

## 4 DISCUSSION

### 4.1 Key findings

This multicountry, mixed‐methods evaluation demonstrated that the proposed LCG design is feasible to use in routine clinical settings. An international sample of 136 doctors, midwives, and nurses from 12 hospitals were trained to use the LCG, and applied it in 1226 low‐risk women. In total, 91.6% of women had a spontaneous vaginal birth and there was an intrapartum stillbirth rate of 1.63 per 1000 births. Practitioners were highly satisfied with the LCG when using it to monitor women in labor. They highlighted it was a coherent and streamlined tool that prompted them to make more precise, accurate documentation during labor, which supported critical thinking in labor care. This made them more responsive to women’s unique needs and facilitating clinical handover. Practitioner experiences became more positive with practice, and they reported that the LCG improved the quality of care provided to women.

We also aimed to identify areas where the LCG design could be improved. Usability evaluation is performed to provide real‐world data on product performance so it can be optimized.^5^, ^14^ Improving the LCG’s design and functionality can in turn improve its acceptance by health care practitioners and reduce misuse.^5^ We interpreted the usability scores (using an adaptation of the validated System Usability Scale) to mean that design refinements could further improve user experience. A major finding was the need to increase the cell size in which regular assessments are documented—practitioners indicated this would make it easier to complete and interpret in busy labor wards. Lower satisfaction scores for some LCG sections (particularly birth outcomes and monitoring of baby) also indicates areas where design can be improved. Usability scores were affected by 45.5% of practitioners indicating that they needed to “learn a lot of things before I could get going with the LCG”. This is possibly related to the training required to use the LCG, or perhaps labor management skills more broadly. This was also reflected in the qualitative findings around the need for practical training to support LCG introduction and implementation. Findings from this evaluation have also informed the improvement of the LCG manual and training materials. Effective initial and ongoing training is essential to ensuring not only that labor observations are performed in a timely manner, but also that the appropriate actions are taken when abnormal observations are identified, including seeking senior help where appropriate.

Practitioners identified several challenges to using LCG that are common to the use of any partograph. A 2014 systematic review on partograph use in low‐income and middle‐income countries found that while the partograph was generally seen as a useful tool for monitoring labor, its use was often perceived as time‐consuming.^13^ Similarly, participating practitioners highlighted that using LCG can be challenging in settings with insufficient numbers of staff and that these pressures can contribute to avoidable errors. Some themes emerging from the qualitative component reflect that not all practitioners are able (or accustomed) to implementing WHO’s full package of intrapartum care recommendations. For example, practitioners described their facility as unable to accommodate labor companions without additional space, privacy measures, or updated facility policies. Furthermore, broader, more systemic barriers identified by practitioners (such as insufficient staff and lack of essential equipment) will negatively affect on the use of any labor monitoring tool.

These findings echo the 2017 realist review by Bedwell et al^12^ that showed that to use a partograph effectively, practitioners need essential equipment (sphygmomanometers, thermometers, and fetal stethoscopes); clear hospital policies on correct partograph use; effective supervision; and regular refresher training. The design of the LCG (or any labor monitoring tool) alone cannot address these broader systemic issues, which require dedicated efforts on quality improvement, infrastructure development, and, in some instances, policy reform and increased staff. Implementation research on how evidence‐based intrapartum care packages can be effectively adopted into routine care in limited‐resource settings is needed. Ideally, these efforts are championed by national or health facility leadership and accompanied by the necessary supplies, training, and enabling environments to ensure practitioners can monitor labor consistently and take prompt action when needed. Some comments from practitioners reflected context‐specific routine practices that may not apply to all settings (eg, that HIV status or blood type should be routinely documented on the tool), which are best addressed through adaptation at national or facility levels.

In some instances, findings from Argentina differed from other sites, including lower levels of satisfaction. These practitioners had on average more years of experience in labor wards compared with practitioners from other countries. This may have led to higher confidence in their usual clinical practice, and less courtesy bias when rating their experience. In addition, Argentinean practitioners reported working in well‐equipped environments with detailed labor records, which could have contributed to greater satisfaction with their usual practice. Furthermore, Argentina was the only study site that used a translated tool and training materials, whose uptake may require cultural adaptation.

Strengths of this project include a robust, mixed‐methods design conducted using a standard protocol across multiple countries and care settings, with a diverse group of practitioners. One limitation was that the prevailing intrapartum care practices (and adapted partograph designs) varied across sites—practitioners sometimes expressed a preference for their customary practice. Another limitation was that not all WHO‐recommended interventions (particularly labor companionship and pain management) were practiced in all hospitals, leading practitioners to highlight that they were unable to use these interventions when prompted by the LCG. In this sense, the LCG is highlighting deficiencies in routine care that need to be acted on by health system managers.

## 5 CONCLUSIONS

The new LCG was feasible to use in different hospitals and countries with variable intrapartum care practices. Although practitioners were highly satisfied, they identified some areas where the LCG design could be improved to enhance usability. As a redesigned WHO partograph, the LCG has the potential to promote woman‐centered care and continuous assessment and decision‐making throughout labor. Implementing the LCG should be accompanied by the necessary initial and ongoing training, and supportive supervision, and strategies to promote an enabling environment for practitioners to use LCG efficiently. This includes ensuring essential equipment is available, and updating facility policies on effective intrapartum interventions.

## Supporting information

SupFile S1

SupFile S2

SupFile S3

Table S1

Table S2

Table S3

Table S4

## Data Availability

The data that support the findings of this study are available from the corresponding author upon reasonable request.
