# Supplementary material for: Usability, acceptability, and feasibility of the World Health Organization Labour Care Guide: A mixed‐methods, multicountry evaluation
Source: Birth. 2020 Nov 22;48(1):66–75. doi: 10.1111/birt.12511 (PMC8246537; doi:10.1111/birt.12511)
Supplement: Supplementary file 1 — SupFile S1 [file BIRT-48-66-s004.pdf]

WHO LABOUR CARE GUIDE

Hospital ID **Participant ID** **Parity** **Labour onset** **Active labour diagnosis [Date** **Time** **]**

Ruptured membranes [Date **Time** **]** **Risk factors**

Section	Item	ALERT	1	2	3	4	5	6	7	8	9	10	11	12	13	14	15	16	17	18	19	20	Second stage 1	Second stage 2	Second stage 3
	Time		:	:	:	:	:	:	:	:	:	:	:	:	:	:	:	:	:	:	:	:	:	:	:
	Hours		1	2	3	4	5	6	7	8	9	10	11	12	13	14	15	16	17	18	19	20	1	2	3
		ALERT	ACTIVE FIRST STAGE																				SECOND STAGE		
SUPPORTIVE CARE	Companion	N																							
	Pain relief	N																							
	Oral fluid	N																							
	Posture	SP																							
BABY	Baseline FHR	<110, ≥160																							
	FHR deceleration	L																							
	Amniotic fluid	M+++, B																							
	Fetal position	OP, OT																							
	Caput	+++																							
	Moulding	+++																							
WOMAN	Pulse	<60, ≥120																							
	Systolic BP	<80, ≥140																							
	Diastolic BP	≥90																							
	Temperature ºC	<35.0, ≥ 37.5																							
	Urine	P++, A++																							
LABOUR PROGRESS	Contractions per 10 min	≤2, >5																							
	Duration of contractions	<20, >60																							
	Cervix [Plot X] 10																								
	Cervix [Plot X] 9	≥ 2h																							
	Cervix [Plot X] 8	≥ 2.5h																							
	Cervix [Plot X] 7	≥ 3h																							
	Cervix [Plot X] 6	≥ 5h																							
	Cervix [Plot X] 5	≥ 6h																							
	Descent [Plot O] 5																								
	Descent [Plot O] 4																								
	Descent [Plot O] 3																								
	Descent [Plot O] 2																								
	Descent [Plot O] 1																								
	Descent [Plot O] 0																								
MEDICATION	Oxytocin (U/L, drops/min)																								
	Medicine																								
	IV fluid																								
SHARED DECISION-MAKING	ASSESSMENT																								
	PLAN																								
	INITIALS																								

In active first stage, plot 'X' to record cervical dilatation. Alert triggered when lag time for current cervical dilatation is exceeded with no progress. In second stage, insert 'P' to indicate when pushing begins.

NOT FOR CLINICAL USE

NOT FOR CLINICAL USE

NOT FOR CLINICAL USE

INSTRUCTIONS: CIRCLE ANY OBSERVATION MEETING THE CRITERIA IN THE 'ALERT' COLUMN, ALERT THE SENIOR MIDWIFE OR DOCTOR AND RECORD THE ASSESSMENT AND ACTION TAKEN.

Abbreviations: **Y –** Yes, **N –** No, **D –** Declined, **U –** Unknown, **SP** – Supine, **MO** – Mobile, **E –** Early, **L –** Late, **V –** Variable, **I –** Intact, **C –** Clear, **M –** Meconium, **B –** Blood, **OA –** Occiput-anterior, **OP –** Occiput-posterior, **OT –** Occiput-transverse, **P –** Protein, **A –** Acetone

Mode of birth **Blood loss** **Neonatal status** **Apgar score at 5 minutes** **Birthweight**

DISCLAIMER: THIS VERSION OF THE WHO LABOUR CARE GUIDE IS ONLY FOR USE DURING THE PILOT-TEST "EVALUATING THE WHO LABOUR CARE GUIDE IN CLINICAL SETTINGS".
