# Supplementary material for: Usability, acceptability, and feasibility of the World Health Organization Labour Care Guide: A mixed‐methods, multicountry evaluation
Source: Birth. 2020 Nov 22;48(1):66–75. doi: 10.1111/birt.12511 (PMC8246537; doi:10.1111/birt.12511)
Supplement: Supplementary file 2 — SupFile S2 [file BIRT-48-66-s003.docx]

**FGD guide coversheet**

Moderator ID __ __ __ __ FGD number __ __

Note-taker ID __ __ __ __ FGD date __ __ /__ __ / 1 9

Country code __ __ __ ^D D M M Y Y^

Facility code __ __ **Start time** __ __ : __ __ am / pm

**End time** __ __ : __ __ am / pm

**Participant characteristics**

| **Participant #  or pseudonym** | **Age group** (circle) | **Cadre** (circle) | **Years’ experience on labour ward** (circle) |
| --- | --- | --- | --- |
| Participant 1 | <30 30-45 >45 | Doctor Midwife  Other (specify): | <5 5–10 11–20 20+ |
| Participant 2 | <30 30-45 >45 | Doctor Midwife  Other (specify): | <5 5–10 11–20 20+ |
| Participant 3 | <30 30-45 >45 | Doctor Midwife  Other (specify): | <5 5–10 11–20 20+ |
| Participant 4 | <30 30-45 >45 | Doctor Midwife  Other (specify): | <5 5–10 11–20 20+ |
| Participant 5 | <30 30-45 >45 | Doctor Midwife  Other (specify): | <5 5–10 11–20 20+ |
| Participant 6 | <30 30-45 >45 | Doctor Midwife  Other (specify): | <5 5–10 11–20 20+ |
| Participant 7 | <30 30-45 >45 | Doctor Midwife  Other (specify): | <5 5–10 11–20 20+ |
| Participant 8 | <30 30-45 >45 | Doctor Midwife  Other (specify): | <5 5–10 11–20 20+ |
| Participant 9 | <30 30-45 >45 | Doctor Midwife  Other (specify): | <5 5–10 11–20 20+ |
| Participant 10 | <30 30-45 >45 | Doctor Midwife  Other (specify): | <5 5–10 11–20 20+ |

**Data management record**

| **Item** | **Response** | **Date** (DD/MM/YY) |
| --- | --- | --- |
| Completed endline questionnaires collected by (ID, initials) |  |  |
| Completed mock Guide photographed by (ID, initials) |  |  |
| Audio recorded by (recorder ID, initials) |  |  |
| Transcription prepared by (ID, initials) |  |  |
| Translation prepared by (ID, initials) |  |  |
| Transcript, field notes, photograph of mock Guide submitted via RedCAP by (ID, initials) |  |  |

***Set-up:***

*A. Materials to prepare in advance:*

- *Audio recorder*
- *12 pens*
- *12 blank copies of the Labour Care Guide*
- *2 large pieces of paper. Each large piece of paper has a simplified template of the Labour Care Guide drawn on it in advance (this is the ‘mock Guide’) – see template on the following page*
- *Name tags or labels, numbered 1-10 or with space for participants to write a confidential nickname*

*B. Setting up the space:*

*The FGD should be conducted in a place where nobody can hear the discussion. There should be space (e.g. on a table, a wall, or the floor) for the participants to work together as a group to draw and write on the mock Guide.*

***Conducting the FGD:***

***Step 1:*** *While participants are arriving, please either ask them to nominate a pseudonym or assign them a participant number, and then give them a name tag or label. You may also wish to ask them to complete the Endline Questionnaire. You may also ask them information that will allow you to complete Table 1 on the FGD guide coversheet (age group; cadre; years’ experience on labour ward; pseudonym or participant number).*

***Step 2:*** *Introduce yourself to the group. Describe the purpose of the FGD and how information will be used. Obtain verbal consent, including consent to audio record the discussion. Explain the ground rules for the discussion.*

***Step 3:***  *Assign each participant a participant number (1-10) or ask them to create a pseudonym (a nickname that is different from their real name), and provide the matching label or name tag for them to wear during the FGD. Please instruct participants to refer to each other by their numbers/pseudonyms, and to say their number/ pseudonym each time they speak. This will allow us to maintain participants’ anonymity.*

***Step 4:***  *Conduct the FGD. Please ensure that all participants state their participant number/pseudonym each time they speak. Please remember to audio record the discussion, including all discussion during the activity (Section D in the discussion guide).*

***Step 5:*** *At the end of the FGD, photograph the completed mock Guide, which the participants have annotated. Ensure that the photograph captures the full diagram, is light enough, and is legible.*

***Step 6****: If participants have not already completed the Endline Questionnaire, and you have not already completed Table 1, please do so now. Complete the rest of the FGD guide coversheet and store it securely.*

***Template for Mock Guide:***

| **WHO LABOUR CARE GUIDE** | | | | |
| --- | --- | --- | --- | --- |
| **Pregnancy information** (parity, labour onset, active labour diagnosis, ruptured membranes, risk factors) | | | | |
|  |  | Time |  |  |
|  |  |  |  |  |
|  |  | **ALERT** | **ACTIVE FIRST STAGE** | **SECOND STAGE** |
| **Supportive care** |  |  |  |  |
| **Baby** |  |  |  |  |
| **Woman** |  |  |  |  |
| **Labour progress** | Contractions |  |  |  |
|  | Cervix |  |  |  |
|  | Descent |  |  |  |
| **Medication** |  |  |  |  |
| **Shared decision-making** | Assessment | |  |  |
|  | Plan |  |  |  |
| Initials |  |  |  |  |
|  | | | | |
| **INSTRUCTIONS** for completing the LCG | | | | |
| **Birth and neonate information** (mode of birth, blood loss, neonatal status, 5min Apgar, birthweight) | | | | |

**Facilitator’s welcome, introduction and instructions to participants**

Welcome and thank you for taking part in this focus group. You have been asked to participate as your point of view is important. I realize you are busy and I appreciate your time.

This focus group discussion is designed to assess your current thoughts and feelings about the WHO Labour Care Guide. The focus group discussion will take around 90 minutes, and no more than two hours. May I tape the discussion to facilitate its recollection? (if yes, switch on the recorder)

**Anonymity:**

Despite being taped, I would like to assure you that the discussion will be anonymous. The tapes will be kept safely in a locked facility until they are transcribed word for word. Once transcribed, the audio recordings will be destroyed. The transcribed notes of the focus group will contain no information that would allow individuals to be linked to specific statements. You should try to answer and comment as accurately and truthfully as possible. I and the other focus group participants would appreciate it if you would refrain from discussing the comments of other group members outside the focus group. If there are any questions or discussions that you do not wish to answer or participate in, you do not have to do so; however please try to answer and be as involved as possible.

**Ground rules:**

- The most important rule is that only one person speaks at a time. There may be a temptation to jump in when someone is talking but please wait until they have finished.
- There are no right or wrong answers
- You do not have to speak in any particular order
- When you do have something to say, please do so. There are many of you in the group and it is important that I obtain the views of each of you
- You do not have to agree with the views of other people in the group
- Please remember to state your participant number each time you speak
- Does anyone have any questions? (answers).
- Does anyone object to participation? You are free to leave if you wish to do so. (answers).
- OK, let’s begin

**A. Feasibility and acceptability of using the WHO Labour Care Guide in clinical settings (approx. 20 minutes)**

Please take a moment to think about your experience with using the Labour Care Guide in your labour ward. We are interested in any negative experiences, as well as positive experiences, because all of this information is important.

1. What did the Labour Care Guide help you to do? Did using the Labour Care Guide make it more difficult to do anything that you would normally do?
2. What was easy about using the Labour Care Guide? What was challenging about using it?
3. What did you enjoy about using the Labour Care Guide? What was not enjoyable about using it?

*Use the following probes if needed:*

- How did the Labour Care Guide influence the **observations** you took during labour?
- Did the Labour Care Guide inform your **decision-making** during labour? Can you share any examples of cases where the Labour Care Guide informed your decision-making?
- How did the Labour Care Guide impact (support or hinder) **communication** between you and other clinical staff, e.g. during handover?
- **When** were you able to complete the Labour Care Guide?

**B. Barriers to using the WHO Labour Care Guide in clinical settings (approx. 15 minutes)**

Let’s take a moment to think around what might prevent or hinder labour ward staff from using the Labour Care Guide. Based on your own experiences with the Labour Care Guide, as well as your understanding of the needs of labour ward staff, and the work environment of the labour ward.

1. Who would like to share their views on what might make it difficult for labour ward staff to routinely use the Labour Care Guide?
2. Were there some times when it simply wasn’t feasible to use the Labour Care Guide? (This is ok!) How about times when it was really challenging to use the Labour Care Guide? Can you tell us about these times? Did others have similar experiences?
3. Are there times when you made decisions that were different from what the Labour Care Guide recommended?

*Use the following probes if needed:*

- Do you think the Labour Care Guide adds to your **workload**, or reduces your workload, or has no impact?
- How did the Labour Care Guide align with other documentation and other processes used to monitor labour? Was there any **duplication** of documents, or replication of tasks?
- Does the availability of **equipment** (such as sphygmomanometers, thermometers and fetal stethoscopes) affect how easy or difficult it is to complete the Labour Care Guide?
- How easy or difficult was it to take any **actions** that were recommended through the Labour Care Guide? *[Facilitation note: ensure this does not become a broad discussion around health system challenges.]*

**C. Facilitators to using the WHO Labour Care Guide in clinical settings (approx. 15 minutes)**

Let’s take a moment to think around what might help labour ward staff to use the Labour Care Guide routinely, based on your knowledge of what helps a labour ward to run well.

1. What do you think would help staff to use the Labour Care Guide, consistently, in routine practice, in the future?
2. Were there times when it was particularly easy to use the Labour Care Guide? Can you tell us about these times? Did others have similar experiences?

*Use the following probes if needed:*

- Can you recall any examples of **teamwork** in using the Labour Care Guide? Did you find this helpful?
- Did you receive any **feedback** on completed Labour Care Guides? Was this helpful? Who provided this feedback?
- Was **supervision** available for completing the Labour Care Guide? Did you find this helpful?
- This project has provided guidelines for completing the Labour Care Guide. Beyond this project, what do you think the role of **facility policies** could be in supporting use of the Labour Care Guide?
- Based on your experience, what support from **colleagues, including supervisors**, do you think would be needed to help you routinely use the Labour Care Guide?

**D. Improvements to the WHO Labour Care Guide (approx. 20 minutes)**

We’d like to hear your views on how the Labour Care Guide itself could be improved. You are now the global experts in using the Guide, and your opinions are really valuable.

*[Bring out the large pieces of paper with the simplified template of the Labour Care Guide, and give each participant a pen & a blank A4 copy of the Labour Care Guide. If the group discussion has been running well, then the whole group can work together on one piece of paper. However, if one or more of the participants has dominated the discussion, then divide the participants into two smaller groups and include the more dominant participants in one group, and the quieter participants in the other group. This way, all participants will have a better opportunity to contribute to the activity.]*

Here we have a large piece of paper that sets out the different sections of the Labour Care Guide. I will also give each of you a blank copy of the Labour Care Guide, and a pen. This is your opportunity to suggest any changes that could be made to the Labour Care Guide. Let’s discuss this together as a group, and once we have agreed on some potential changes, let’s draw or write our suggested changes on the big piece of paper. We can also write any comments about the Labour Care Guide on the big piece of paper. Please do not write on the small Labour Care Guides – they are for your reference only. We will photograph the big piece of paper at the end of the FGD, so we need to make sure that what we write is clear.

1. Who would like to make some suggestions for change? *[Facilitation note: ensure that participants discuss what to write or draw on the mock Guide, and ensure that participants’ discussion is recorded. If necessary, the facilitator may need to describe what the participants are writing, so that it is captured on the audio-recording. The mock Guide will be photographed at the end of the FGD.]*

*Use the following probes if needed:*

- Are any parts of the Labour Care Guide **difficult** to understand, or difficult to complete? Can you explain what is difficult?
- What are your thoughts on the **structure** of the Labour Care Guide?
- Is there anything that needs to be **removed** from the Labour Care Guide?
- Is there anything you feel should be **included** in the Labour Care Guide and is not?

**E. Improvements to the WHO Labour Care Guide training (approx. 10 minutes)**

Let’s think back to how the Labour Care Guide was introduced to you in the training, and then the process of beginning to use the Guide in practice.

1. How do you think the training could be improved?

*Use the following probes if needed:*

*High priority topics:*

- How **prepared** did you feel when you first started using the Labour Care Guide?
- What **helped you the most** in learning to use the Labour Care Guide, either during or after the training?
- What was not covered in the training that you **learnt through using** the Labour Care Guide?
- What should be included in **future trainings** for labour ward staff on using the Labour Care Guide?
- Which **groups/cadres** of labour ward staff should be trained on using the Labour Care Guide?

*Lower priority topics (can be discussed more quickly):*

- What do you think about the **length** of the training – too long or too short?
- What do you think about the degree of **difficulty** of the training – too simple or too complicated?
- What did you think of the **Provider Training Manual** – did you like it or dislike it? How could it be improved?
- Do you think future training on using the Labour Care Guide could be **self-directed**? Do you think it could be delivered **electronically**?
- How frequently would you like to receive **refresher training** in using the Labour Care Guide?

**Conclusion**

Is there anything that you think I may have misunderstood today? Is there anything that you would like to add before we conclude our discussion?

Thank you for participating. This has been a very successful discussion. Your opinions are a valuable asset to this project and will assist the use of the Labour Care Guide by other providers.

If there is anything you are unhappy with or wish to complain about, please contact the local Site Lead or speak to me later. I would like to remind you that any comments featuring in this report will be anonymous.

***If participants have not already completed the Endline Questionnaire, please ask them to do so now.***

***If you have not already worked with participants to complete Table 1, please do so now.***

***Please complete the rest of the FGD guide coversheet and store it securely.***

**THANK YOU – AND CONGRATULATIONS ON COMPLETING THIS IMPORTANT WORK!**
