# Supplementary material for: Usability, acceptability, and feasibility of the World Health Organization Labour Care Guide: A mixed‐methods, multicountry evaluation
Source: Birth. 2020 Nov 22;48(1):66–75. doi: 10.1111/birt.12511 (PMC8246537; doi:10.1111/birt.12511)
Supplement: Supplementary file 3 — SupFile S3 [file BIRT-48-66-s005.docx]

**Supplementary File S3. System Usability Score**

Participating providers were invited to complete a paper questionnaire after enrolment had concluded. The questionnaire included an adaptation of the System Usability Scale (SUS), which had been tailored to the Labour Care Guide. The SUS is a valid, reliable and widely used measure for assessing usability, consisting of a 10-item questionnaire with a Likert scale (five response options of strongly agree to strongly disagree).

The SUS score was calculated according to a standardised process, as recommended by the developers.

- Likert scale values were assigned values of 1 (strongly disagree) to 5 (strongly agree)
- These values were converted to a score out of 4:
  - Minus 1 from the scores of the odd-numbered questions
  - The value of even-numbered questions was subtracted from 5 to get new value.
- The score was multiplied by 2.5 to a score are converted to a score out of 100

The table below provides an example. Similar to other authors, a score of 70 or greater was pre-defined as “good usability”. SUS scores should not be interpreted as percentages.

| Question | Example answer | Example score |
| --- | --- | --- |
| 1. I think that I would like to use the Labour Care Guide frequently. | *Strongly Agree (=5)* | 4 |
| 1. I found the Labour Care Guide unnecessarily complex. | *Strongly Disagree (=1)* | 4 |
| 1. I thought Labour Care Guide was easy to use. | *Agree (=4)* | 3 |
| 1. I think that I would need assistance to be able to use the Labour Care Guide. | *Disagree (=2)* | 3 |
| 1. I found the various functions of the Labour Care Guide were well integrated. | *Strongly Agree (=5)* | 4 |
| 1. I thought there was too much inconsistency in the Labour Care Guide. | *Strongly Disagree (=1)* | 4 |
| 1. I would imagine that most people like me would learn to use the Labour Care Guide very quickly. | *Agree (=4)* | 3 |
| 1. I found the Labour Care Guide very cumbersome/awkward to use. | *Disagree (=2)* | 3 |
| 1. I felt very confident using the Labour Care Guide. | *Strongly Agree (=5)* | 4 |
| 1. I needed to learn a lot of things before I could get going with the Labour Care Guide. | *Strongly Disagree (=1)* | 4 |
| *Score (out of 40)* |  | 36 |
| *Score (out of 100)* |  | 90 |
