# Supplementary material for: Usability, acceptability, and feasibility of the World Health Organization Labour Care Guide: A mixed‐methods, multicountry evaluation
Source: Birth. 2020 Nov 22;48(1):66–75. doi: 10.1111/birt.12511 (PMC8246537; doi:10.1111/birt.12511)
Supplement: Supplementary file 4 — Table S1 [file BIRT-48-66-s007.docx]

**Supplementary Table S1. Characteristics of participating hospitals**

| **Country** | **Argentina** | | **India** | | **Kenya** | | **Malawi** | | **Nigeria** | | **Tanzania** | |
| --- | --- | --- | --- | --- | --- | --- | --- | --- | --- | --- | --- | --- |
| **Hospital number** | **1** | **2** | **3** | **4** | **5** | **6** | **7** | **8** | **9** | **10** | **11** | **12** |
| Setting | Urban | Urban | Urban | Urban | Urban | Urban | Urban | Rural | Urban | Urban | Urban | Semi-Urban |
| Obstetrician available on site (24/7) | Yes | Yes | Yes | Yes | Yes | Yes | Yes | On call | Yes | Yes | Yes | On call |
| Total births in 2018 (singletons and multiples) | 4,663 | 1,106 | 4,232 | 12,879 | 10,750 | 3,405 | 11,191 | 7,245 | 3,283 | 3,945 | 5,774 | 7,382 |
| CS rate (2018) (%) | 32% | 30% | 51% | 40% | 45% | 46% | 30% | 20% | 25% | 21% | 35% | 15% |
| Total stillbirths^[[1]](#footnote-1)^ and stillbirth rate in 2018 | 24  (0.51%) | 5  (0.45%) | 105  (2.48%) | 260 (2.02%) | 606 (5.64%) | 59  (1.73%) | 230 (2.06%) | 139 (1.92%) | 185 (5.64%) | 90  (2.23%) | 121 (2.10%) | 110  (1.49%) |
| Is a partograph in routine use at this hospital | Yes | Yes | Yes | Yes | Yes | Yes | Yes | Yes | Yes | Yes | Yes | Yes |
| Care providers attending low-risk births in this hospital | Midwives  Residents Obstetricians | Midwives  Residents Obstetricians | Staff Nurses Residents Consultants | Nurses  Junior doctors | Nurses  Midwives  Doctors | Nurses  Midwives  Doctors | Nurses  Midwives  Doctors | Nurses  Midwives  Doctors | Nurses  Midwives  Doctors | Nurses  Midwives  Doctors | Nurses  Midwives  Doctors | Nurses  Midwives  Doctors |
| Comprehensive emergency obstetric care available^[[2]](#footnote-2)^ | Yes | Yes | Yes | Yes | Yes | Yes | Yes | Yes | Yes | Yes | Yes | Yes |

1. As defined by that health facility [↑](#footnote-ref-1)
2. Based on signal functions, hospitals can: parenteral antibiotics, administer uterotonic drugs (i.e. parenteral oxytocin), administer parenteral anticonvulsants for preeclampsia and eclampsia (i.e. magnesium sulfate), perform manual removal of the placenta, perform removal retained products (e.g. manual vacuum extraction, dilation and curettage), perform assisted vaginal delivery (e.g. vacuum extraction, forceps delivery), perform basic neonatal resuscitation (e.g. with bag and mask), perform surgery (e.g. caesarean section), perform blood transfusion [↑](#footnote-ref-2)
