# Supplementary material for: Usability, acceptability, and feasibility of the World Health Organization Labour Care Guide: A mixed‐methods, multicountry evaluation
Source: Birth. 2020 Nov 22;48(1):66–75. doi: 10.1111/birt.12511 (PMC8246537; doi:10.1111/birt.12511)
Supplement: Supplementary file 5 — Table S2 [file BIRT-48-66-s001.docx]

**Supplementary Table 2. Provider’s overall assessment following Labour Care Guide training**

|  | | **Argentina** | | **India** | | **Kenya** | | **Malawi** | | **Nigeria** | | **Tanzania** | | **Total** | |
| --- | --- | --- | --- | --- | --- | --- | --- | --- | --- | --- | --- | --- | --- | --- | --- |
|  |  | **N** | **%** | **N** | **%** | **N** | **%** | **N** | **%** | **N** | **%** | **N** | **%** | **N** | **%** |
| Total | | **32** | **100%** | **23** | **100%** | **20** | **100%** | **20** | **100%** | **20** | **100%** | **21** | **100%** | **136** | **100%** |
| Overall, I found the training in using the Labour Care Guide to be helpful | strongly agree | 8 | 25.0% | 14 | 60.9% | 12 | 54.5% | 12 | 52.2% | 15 | 75.0% | 11 | 47.8% | 72 | 50.3% |
|  | agree | 18 | 56.3% | 9 | 39.1% | 10 | 45.5% | 11 | 47.8% | 5 | 25.0% | 11 | 47.8% | 64 | 44.8% |
|  | neither agree nor disagree | 5 | 15.6% | 0 | 0.0% | 0 | 0.0% | 0 | 0.0% | 0 | 0.0% | 1 | 4.3% | 6 | 4.2% |
|  | disagree | 1 | 3.1% | 0 | 0.0% | 0 | 0.0% | 0 | 0.0% | 0 | 0.0% | 0 | 0.0% | 1 | 0.7% |
|  | strongly disagree | 0 | 0.0% | 0 | 0.0% | 0 | 0.0% | 0 | 0.0% | 0 | 0.0% | 0 | 0.0% | 0 | 0.0% |
| I feel I could use the Labour Care Guide in labour ward tomorrow | strongly agree | 3 | 9.4% | 13 | 56.5% | 9 | 42.9% | 10 | 43.5% | 9 | 45.0% | 9 | 39.1% | 53 | 37.3% |
|  | agree | 15 | 46.9% | 10 | 43.5% | 5 | 23.8% | 12 | 52.2% | 9 | 45.0% | 13 | 56.5% | 64 | 45.1% |
|  | neither agree nor disagree | 10 | 31.3% | 0 | 0.0% | 6 | 28.6% | 1 | 4.3% | 2 | 10.0% | 1 | 4.3% | 20 | 14.1% |
|  | disagree | 3 | 9.4% | 0 | 0.0% | 1 | 4.8% | 0 | 0.0% | 0 | 0.0% | 0 | 0.0% | 4 | 2.8% |
|  | strongly disagree | 1 | 3.1% | 0 | 0.0% | 0 | 0.0% | 0 | 0.0% | 0 | 0.0% | 0 | 0.0% | 1 | 0.7% |
