# Supplementary material for: Usability, acceptability, and feasibility of the World Health Organization Labour Care Guide: A mixed‐methods, multicountry evaluation
Source: Birth. 2020 Nov 22;48(1):66–75. doi: 10.1111/birt.12511 (PMC8246537; doi:10.1111/birt.12511)
Supplement: Supplementary file 6 — Table S3 [file BIRT-48-66-s006.docx]

**Supplementary Table 3. Provider experiences with using the Labour Care Guide at each birth**

|  | **Country** | | | | | | **Professional role** | | | **Provider experience with LCG** | | **Maternal parity** | |
| --- | --- | --- | --- | --- | --- | --- | --- | --- | --- | --- | --- | --- | --- |
|  | **Argentina**  **N**  **(%)** | **India**  **N**  **(%)** | **Kenya**  **N**  **(%)** | **Malawi**  **N**  **(%)** | **Nigeria**  **N**  **(%)** | **Tanzania**  **N**  **(%)** | **Doctor**  **N**  **(%)** | **Midwife N**  **(%)** | **Nurse N**  **(%)** | **<=5 births**  **N**  **(%)** | **>5 births N**  **(%)** | **0**  **N**  **(%)** | **1+**  **N**  **(%)** |
| **Total** | **203**  **(100)** | **200**  **(100)** | **201**  **(100)** | **201**  **(100)** | **200**  **(100)** | **220**  **(100)** | **368**  **(100)** | **819**  **(100)** | **38**  **(100)** | **638**  **(100)** | **587**  **(100)** | **532**  **(100)** | **693**  **(100)** |
| **I was able to use the LCG in managing this labour and childbirth** |  |  |  |  |  |  |  |  |  |  |  |  |  |
| strongly agree | 21  (10.3) | 126 (63.0) | 114 (56.7) | 95  (47.3) | 102 (51.0) | 122 (55.5) | 205  (55.7) | 372  (45.4) | 3  (7.9) | 279  (43.7) | 301  (51.3) | 249  (46.8) | 331  (47.8) |
| agree | 142 (70.0) | 74 (37.0) | 84 (41.8) | 103 (51.2) | 96  (48.0) | 98  (44.5) | 154  (41.8) | 408  (49.8) | 35  (92.1) | 326  (51.1) | 271  (46.2) | 264  (49.6) | 333  (48.1) |
| neither agree nor disagree | 38  (18.7) | 0  (0) | 3  (1.5) | 3  (1.5) | 2  (1.0) | 0  (0) | 9  (2.4) | 37  (4.5) | 0  (0) | 31  (4.9) | 15  (2.6) | 18  (3.4) | 28  (4.0) |
| disagree | 2  (1.0) | 0  (0) | 0  (0) | 0  (0) | 0  (0) | 0  (0) | 0  (0) | 2  (0.2) | 0  (0) | 2  (0.3) | 0  (0) | 1  (0.2) | 1  (0.1) |
| strongly disagree | 0  (0) | 0  (0) | 0  (0) | 0  (0) | 0  (0) | 0  (0) | 0  (0) | 0  (0) | 0  (0) | 0  (0) | 0  (0) | 0  (0) | 0  (0) |
| **I was able to complete the LCG correctly** |  |  |  |  |  |  |  |  |  |  |  |  |  |
| strongly agree | 17  (8.4) | 101 (50.5) | 109 (54.2) | 80  (39.8) | 68  (34.0) | 120 (54.5) | 158  (42.9) | 336  (41.0) | 1  (2.6) | 236  (37.0) | 259  (44.1) | 206  (38.7) | 289  (41.7) |
| agree | 135 (66.5) | 99 (49.5) | 86 (42.8) | 113 (56.2) | 126 (63.0) | 100 (45.5) | 199  (54.1) | 424  (51.8) | 36  (94.7) | 352  (55.2) | 307  (52.3) | 298  (56.0) | 361  (52.1) |
| neither agree nor disagree | 41  (20.2) | 0  (0) | 6  (3.0) | 4  (2.0) | 6  (3.0) | 0  (0) | 10  (2.7) | 46  (5.6) | 1  (2.6) | 39  (6.1) | 18  (3.1) | 21  (3.9) | 36  (5.2) |
| disagree | 10  (4.9) | 0  (0) | 0  (0) | 4  (0.2) | 0  (0) | 0  (0) | 1  (0.3) | 13  (1.6) | 0  (0) | 11  (1.7) | 3  (0.5) | 7  (1.3) | 7  (1.0) |
| strongly disagree | 0  (0) | 0  (0) | 0  (0) | 0  (0) | 0  (0) | 0  (0) | 0  (0) | 0  (0) | 0  (0) | 0  (0) | 0  (0) | 0  (0) | 0  (0) |
| **I was satisfied using the LCG in managing this woman’s labour and childbirth** |  |  |  |  |  |  |  |  |  |  |  |  |  |
| strongly agree | 3  (1.5) | 107 (53.5) | 102 (50.7) | 70  (34.8) | 76  (38.0) | 121 (55.0) | 144  (39.1) | 333  (40.7) | 2  (5.3) | 212  (33.2) | 267  (45.5) | 195  (36.7) | 284  (41.0) |
| agree | 114 (56.2) | 93 (46.5) | 93 (46.3) | 112 (55.7) | 116 (58.0) | 99  (45.0) | 198  (53.8) | 394  (48.1) | 35  (92.1) | 347  (54.4) | 280  (47.7) | 289  (54.3) | 338  (48.8) |
| neither agree nor disagree | 81  (39.9) | 0  (0) | 6  (3.0) | 13  (6.5) | 8  (4.0) | 0  (0) | 23  (6.3) | 85  (10.4) | 0  (0) | 71  (11.1) | 37  (6.3) | 42  (7.9) | 66  (9.5) |
| disagree | 5  (2.5) | 0  (0) | 0  (0) | 6  (3.0) | 0  (0) | 0  (0) | 3  (0.8) | 7  (0.9) | 1  (2.6) | 8  (1.3) | 3  (0.5) | 6  (1.1) | 5  (0.7) |
| strongly disagree | 0  (0) | 0  (0) | 0  (0) | 0  (0) | 0  (0) | 0  (0) | 0  (0) | 0  (0) | 0  (0) | 0  (0) | 0  (0) | 0  (0) | 0  (0) |
| **The LCG was helpful in managing this woman’s labour and childbirth** |  |  |  |  |  |  |  |  |  |  |  |  |  |
| strongly agree | 6  (3.0) | 114 (57.0) | 102 (50.7) | 85  (42.3) | 89  (44.5) | 120 (54.5) | 165  (44.8) | 346  (42.2) | 5  (13.2) | 245  (38.4) | 271  (46.2) | 214  (40.2) | 302  (43.6) |
| agree | 117 (57.6) | 84 (42.0) | 96 (47.8) | 99  (49.3) | 104 (52.0) | 100 (45.5) | 179  (48.6) | 390  (47.6) | 31  (81.6) | 316  (49.5) | 284  (48.4) | 274  (51.5) | 326  (47.0) |
| neither agree nor disagree | 72  (35.5) | 2  (1.0) | 3  (1.5) | 11  (5.5) | 7  (3.5) | 0  (0) | 21  (5.7) | 73  (8.9) | 1  (2.6) | 66  (10.3) | 29  (4.9) | 38  (7.1) | 57  (8.2) |
| disagree | 8  (3.9) | 0  (0) | 0  (0) | 6  (3.0) | 0  (0) | 0  (0) | 3  (0.8) | 10  (1.2) | 1  (2.6) | 11  (1.7) | 3  (0.5) | 6  (1.1) | 8  (1.2) |
| strongly disagree | 0  (0) | 0  (0) | 0  (0) | 0  (0) | 0  (0) | 0  (0) | 0  (0) | 0  (0) | 0  (0) | 0  (0) | 0  (0) | 0  (0) | 0  (0) |
| **Overall, I am satisfied with the current design of the LCG** |  |  |  |  |  |  |  |  |  |  |  |  |  |
| strongly agree | 3  (1.5) | 94 (47.0) | 73 (36.3) | 57  (28.4) | 62  (31.0) | 121 (55.0) | 124  (33.7) | 286  (34.9) | 0  (0) | 188  (29.5) | 222  (37.8) | 155  (29.1) | 255  (36.8) |
| agree | 65  (32.0) | 106 (53.0) | 111 (55.2) | 93  (46.3) | 91  (45.5) | 99  (45.0) | 181  (49.2) | 349  (42.6) | 35  (92.1) | 290  (45.5) | 275  (46.8) | 271  (50.9) | 294  (42.4) |
| neither agree nor disagree | 106 (52.2) | 0  (0) | 17  (8.5) | 33  (16.4) | 43  (21.5) | 0  (0) | 52  (14.1) | 145  (17.7) | 2  (5.3) | 118  (18.5) | 81  (13.8) | 83  (15.6) | 116  (16.7) |
| disagree | 28  (13.8) | 0  (0) | 0  (0) | 15  (7.5) | 4  (2.0) | 0  (0) | 11  (3.0) | 36  (4.4) | 0  (0) | 39  (6.1) | 8  (1.4) | 20  (3.8) | 27  (3.9) |
| strongly disagree | 1  (0.5) | 0  (0) | 0  (0) | 3  (1.5) | 0  (0) | 0  (0) | 0  (0) | 3  (0.4) | 1  (2.6) | 3  (0.5) | 1  (0.2) | 3  (0.6) | 1  (0.1) |
