# Supplementary material for: Usability, acceptability, and feasibility of the World Health Organization Labour Care Guide: A mixed‐methods, multicountry evaluation
Source: Birth. 2020 Nov 22;48(1):66–75. doi: 10.1111/birt.12511 (PMC8246537; doi:10.1111/birt.12511)
Supplement: Supplementary file 7 — Table S4 [file BIRT-48-66-s002.docx]

**Supplementary Table S4. Provider’s satisfaction with components of the Labour Care Guide by end of project**

|  | | **N** | **%** |
| --- | --- | --- | --- |
| Total | | **136** | **100%** |
| I am satisfied with the identifying section (section 1) of the Labour Care Guide | strongly agree | 33 | 24.3% |
|  | agree | 74 | 54.4% |
|  | neither agree nor disagree | 13 | 9.6% |
|  | disagree | 16 | 11.8% |
|  | strongly disagree | 0 | 0.0% |
| I am satisfied with the supportive care section (section 2) of the Labour Care Guide | strongly agree | 34 | 25.0% |
|  | agree | 85 | 62.5% |
|  | neither agree nor disagree | 7 | 5.1% |
|  | disagree | 10 | 7.4% |
|  | strongly disagree | 0 | 0.0% |
| I am satisfied with the baby section (section 3) of the Labour Care Guide | strongly agree | 31 | 22.8% |
|  | agree | 63 | 46.3% |
|  | neither agree nor disagree | 22 | 16.2% |
|  | disagree | 18 | 13.2% |
|  | strongly disagree | 2 | 1.5% |
| I am satisfied with the mother section (section 4) of the Labour Care Guide | strongly agree | 38 | 27.9% |
|  | agree | 75 | 55.1% |
|  | neither agree nor disagree | 14 | 10.3% |
|  | disagree | 8 | 5.9% |
|  | strongly disagree | 1 | 0.7% |
| I am satisfied with the labour progress section (section 5) of the Labour Care Guide | strongly agree | 49 | 36.0% |
|  | agree | 59 | 43.4% |
|  | neither agree nor disagree | 17 | 12.5% |
|  | disagree | 11 | 8.1% |
|  | strongly disagree | 0 | 0.0% |
| I am satisfied with the medication section (section 6) of the Labour Care Guide | strongly agree | 37 | 27.2% |
|  | agree | 72 | 52.9% |
|  | neither agree nor disagree | 16 | 11.8% |
|  | disagree | 10 | 7.4% |
|  | strongly disagree | 1 | 0.7% |
| I am satisfied with the shared decision-making section (section 7) of the Labour Care Guide | strongly agree | 30 | 22.1% |
|  | agree | 85 | 62.5% |
|  | neither agree nor disagree | 9 | 6.6% |
|  | disagree | 11 | 8.1% |
|  | strongly disagree | 1 | 0.7% |
| I am satisfied with the birth outcome section (section 8) of the Labour Care Guide | strongly agree | 20 | 14.7% |
|  | agree | 70 | 51.5% |
|  | neither agree nor disagree | 26 | 19.1% |
|  | disagree | 18 | 13.2% |
|  | strongly disagree | 2 | 1.5% |
| Overall, I am satisfied with the current design of the Labour Care Guide | strongly agree | 18 | 13.2% |
|  | agree | 85 | 62.5% |
|  | neither agree nor disagree | 19 | 14.0% |
|  | disagree | 14 | 10.3% |
|  | strongly disagree | 0 | 0.0% |
